# Obesity-Related Adipose Tissue Remodeling in the Light of Extracellular Mitochondria Transfer

**DOI:** 10.3390/ijms23020632

**Published:** 2022-01-06

**Authors:** Simon Lecoutre, Karine Clément, Isabelle Dugail

**Affiliations:** Nutrition et Obesities, Systemic Approaches, NutriOmique, Sorbonne Université, INSERM, F-75013 Paris, France; 23simon.lec@gmail.com (S.L.); karine.clement2@gmail.com (K.C.)

**Keywords:** adipose tissue, mitochondria, metabolism, inflammation, remodeling

## Abstract

Adipose tissue dysfunction is strongly associated with obesity and its metabolic complications such as type 2 diabetes and cardiovascular diseases. It is well established that lipid-overloaded adipose tissue produces a large range of secreted molecules that contribute a pro-inflammatory microenvironment which subsequently disseminates towards multi-organ metabolic homeostasis disruption. Besides physiopathological contribution of adipose-derived molecules, a new paradigm is emerging following the discovery that adipocytes have a propensity to extrude damaged mitochondria in the extracellular space, to be conveyed through the blood and taken up by cell acceptors, in a process called intercellular mitochondria transfer. This review summarizes the discovery of mitochondria transfer, its relation to cell quality control systems and recent data that demonstrate its relevant implication in the context of obesity-related adipose tissue dysfunction.

## 1. Discovery of Extracellular Mitochondria Transfer

Mitochondria are intracellular organelles that orchestrate energy production in eukaryotes; they also orchestrate calcium homeostasis, iron homeostasis and signaling to the execution of apoptosis. As such, they undergo highly dynamic continuous adaptive changes to fulfil power requirements and optimize metabolic function in changing environments. A striking vision of their dynamic nature is illustrated by time-lapse microscopy, showing impressive morphological fission/fusion events and constant remodeling of the intracellular mitochondrial network. Mitochondria are no more a static compartment within the cytoplasm, but instead a mobile plastic entity with multiple connections with other organelles. The concept of mitochondria dynamics has built over the years on examples of mitochondria kinesis in almost every cell type, from neurones where mitochondria move from the cell body along axons and backwards [1], to invasive cancer cells where they traffic to the leading edge to provide local energy [2]. In short-term energy demand, they become associated with lipid droplets to gain efficiency in lipid oxidation [3]. Also striking is the unique process for organelle degradation within lysosomes referred to as mitophagy, in which tagged mitochondria are engulfed into vesicles and conveyed to acidic compartments for subsequent degradation.

In line with numerous examples of mitochondrial dynamics, the idea that mitochondria could travel outside of cell borders is recently emerging. It might be reminiscent of the bacterial ancestral origins of mitochondria, which once colonized eukaryotic cells from the exterior. The first evidence came from the discovery of nanotubular formation as a new mode of intercellular communication, through which exchange of material between connected cells could include mitochondria [4]. Strikingly, it was observed that cells lacking functionally competent mitochondria can capture purified mitochondria from supernatants and that this process promoted cell proliferation [5]. Then, another mode of mitochondria channeling, based on connexin-43 intercellular gap junctions was recognized [6], followed by identification of a third type of organelle extrusion using microvesicle production from a cell donor to a more distant, physically unconnected cell acceptor [7,8]. A recent review provides extensive analysis of data in the literature that originally described mitochondria transfer events in various conditions and cell types [9], summarized in Table 1. It suggests that quite a large diversity exists in the mode of intercellular connection and the type of vesicles that support mitochondria extrusion (exophers, microvesicles, exosomes or autophagosome-like doubled membranes), possibly reflecting different processes at the level of the donor cell, leading to the recently proposed “mitovesicles” as a general denomination [10].

At the molecular level, the process is far from completely elucidated, even if some molecular adaptors required for mitochondrial movement along microtubules have been identified, such as the Miro1 Rho GTPase [11]. Recently, nanotubes formation was reported to require cell to cell surface adhesion molecule Cd38 [12], whereas Mitofusin 2, a GTPase that tethers endoplasmic reticulum to mitochondria was shown to regulate mitochondria transfer between astrocytes [13]. Future identification of main molecular actors involved in the process of cell extrusion will provide powerful tools for modulating mitochondria intercellular exchanges, towards functional investigations.

## 2. Mitochondria Extrusion as a Quality Control Process

Obviously, extracellular mitochondria transfer can be considered a mode of mitochondria clearance, closely linked to quality control status of the donor cell. Mitochondria are subject to tight quality control, enabled by mitochondrial proteases, proteasome-mediated degradation of outer mitochondrial membrane proteins, formation of mitochondrial-derived vesicles and mitophagy [23]. It appears from several studies that mitochondria transfer is more frequent when intracellular pathways for degradation of damaged components are compromised or overwhelmed. For example, large exophers production by *C. elegans* neurons that contain protein aggregates and mitochondria is enhanced following inhibition of chaperone expression, proteasome activity or autophagy [8]. More recently, in a study on neutrophils migration where cell motion is achieved through expansion of retraction fibers that subsequently break and leave behind cellular material attached to the substratum (called migrasomes), it was reported that cell pre-treatment with a respiratory uncoupler led to mitochondria enrichment in migrasomes [24]. This is in accordance with mitochondrial stress being a trigger for organelle extrusion. Importantly, in this study, damaged mitochondria were selectively disposed of, and were found to contribute to overall maintenance of neutrophil quality in vivo. Thus, extracellular mitochondrial transfer has strong potential as a previously unrecognized quality control mechanism to get rid of damaged organelles. Considering that a hallmark of ageing is the accumulation of damaged cell components, it is tempting to speculate that benefits would result from stimulating mitochondria transfer against the decline in cellular regenerative functions. Further, a recent study indicates that mitochondria transfer can be a way for cancer cells to evade cell death caused by autophagy deficiency [14]. However, the links between mitophagy and mitochondrial extrusion might be more complex than a simple alternate response to intracellular degradation blockade. Indeed, in some cases, mitochondria extrusion by mesenchymal stem cells is limited by autophagy inhibition, leading to massive apoptotic response [7].

## 3. Intercellular Mitochondria Exchange Protects against Pathologies

Even if the advantage of ameliorated mitochondria clearance can be expected for donor cells, the largest body of experiments focuses on the beneficial outcomes of incorporating new mitochondria material into a cell recipient. Indeed, fluorescent imaging clearly indicates that extruded mitochondria can be taken up by surrounding cells. Especially, extracellular mitochondria can be avidly taken up by macrophages, reminiscent of their phagocytic functions [7]. Indeed, macrophage phagocytosis of exophers containing defective mitochondria ejected from cardiomyocytes among other cellular contents was reported to support overall cardiomyocyte health [15]. More generally, as immune cells undergo a metabolic switch from an active oxidative metabolism towards glycolysis during inflammation [25], mitochondria uptake could sustain recipient cell potential for oxidative phosphorylation (OXPHOS) by reinforcing the mitochondria pool. As an example, in the response of lung epithelium to acute lipopolysaccharide-mediated injury, ATP production is improved following acquisition of mitochondria from bone-marrow-derived mesenchymal stem cells (MSC), as well as the survival rate of treated mice [6]. Similarly, in a model of acute respiratory distress, mitochondria transfer to alveolar macrophages from MSC improved oxidative phosphorylation and promoted M2 anti-inflammatory differentiation with subsequent reduction in lung inflammation [16]. The importance of mitochondria transfer in the general context of macrophage biology has been recently reviewed in [26].

The potential of mitochondria transfer in neural degenerative processes causing the most frequent diseases in an ageing population (Parkinson’s, Alzheimer’s) has been raised over time. Neurones were described as benefiting from mitochondria transfer from surrounding astrocytes. This was demonstrated using astroglial/neuronal co-culture systems, where internalization of mitochondria by rotenone-injured neurons was associated with reversal of neurodegeneration and axonal pruning [27]. In vivo, upon transient focal cerebral ischemia in mice, astrocytes do release functional mitochondria that are taken up by neurons [17]. These findings suggest a role for mitochondria transfer in neurorecovery after stroke. The scope of the therapeutic potential of mitochondria transfer in neurodegenerative diseases is a rapidly expanding field, covered in detail in [9].

Moreover, because a hallmark of cancer cells is their reprogrammed metabolism and ability to upregulate glycolysis in an aerobic environment known as the Warburg effect, the role of mitochondria transfer in the context of developing tumors is investigated and reviewed in [9]. As a general principle, stimulating OXPHOS is an important component of cancer cell plasticity and a contributor in tumor progression. Additionally, metabolic reprogramming is at the center of the development of chemoresistant phenotypes by cancer cells to evade treatment. In this regard, it is important to consider the influence of cells present in the tumor environment, such as cancer-associated fibroblasts or immune cells that might initiate mitochondria exchange within the tumor.

It was originally believed that the regenerative properties of MSC was supported by their ability to colonize and proliferate at the site of tissue injury, to promote reparation though de novo differentiation from a multipotent phenotype substituting deficient cell types. This idea has evolved as experiments frequently observed a discrepancy between the regenerative benefit of MSC injection and the low frequency of exogenously provided cell engraftment at the site of regeneration. Moreover, studies indicate that the regenerative properties of MSC strongly depend on signaling through metabolites and paracrine factors or delivering extracellular mitochondria to damaged tissue or cells. However, how protection/reparation is conferred to recipient cells by mitochondria transfer is still poorly understood. A simple explanation is that additional oxidative power results from acquisition of new functional mitochondria [5]. Mitochondrial donation by MSCs is a faster and a more economical physiological process than mitochondrial biogenesis, and thus is an efficient means to attenuate disease conditions. However, this might not be the only way additional mitochondria might impact cell function. Platelets can release and transfer mitochondria to MSC [18]. During wound healing, this was found to facilitate angiogenesis, associated with the elevation of mitochondria-derived cytosolic citrate level and subsequent stimulation of fatty acid synthesis [19].

In the field of regenerative medicine, the question of whether mitochondria transfer can overcome the consequences of mitochondrial DNA alteration or mutations by regenerating mitochondrial genome is still poorly investigated. In this line, special attention has been given to Humanin, a mitochondria-encoded protein, that has been shown to mimic the beneficial effects of mitochondria transfer from astrocytes alone or in its mitochondria-associated form [28]. In this study, the phagocytosis of mitochondria or Humanin cell exposure both led to the induction of the transcription factor Peroxisome Proliferation-Activated Receptor gamma (PPARg), illustrating the possibility that closely associated mitochondrial component could contribute, at least partly, to the activity elicited by the entire organelle. Mitochondrial DNA is mainly involved in immunity as a damage-associated molecular pattern (DAMP), and thus it remains to be determined if transfer might modulate cell inflammatory phenotype through mitochondrial DNA.

## 4. Special Focus on Adipose Tissue Mitochondria Status in Obesity and Metabolic Disorders

### 4.1. The Status of Adipose Mitochondria in Obesity

Obesity is a condition of excessive fat accretion, driven by chronic positive energy balance where food intake exceeds caloric expenditure. Since energy homeostasis is at the center of the game, it is not surprising that obesity impacts mitochondria, particularly in adipose tissue, which is the main site for deposition of excess energy as fat. In the context of chronic overnutrition, oxidative stress may be induced by imbalance between anti-oxidant defense and pro-oxidant load generated by reactive oxygen species (ROS) and free radicals. ROS production is largely due to intense breakdown of metabolites in the tri-carboxylic acid (TCA) cycle, which may exceed the capacity of the electron transport chain to assimilate the resulting electrons [29]. Some adaptive pathways exist to minimize carbon burden to TCA, called vent pathways, which can moderate ROS production when activated. Those include nucleocytoplasmic citrate channeling to fuel histone acetylation and fatty acid synthesis, or reduction of carbon entry to TCA by lactate production from pyruvate, or inhibition of glutamine-derived anaplerosis through alpha-ketoglutarate [30].

Main findings on adipose tissue mitochondria status are summarized in the recent review by [31]. Most studies agree in reporting general mitochondrial dysfunction in obesity, dominated by decreased mitochondrial mass, downregulated biological functions and biogenesis. In particular, mtDNA amount and gene expression levels of mitochondria-related pathways are downregulated in co-twins with obesity compared with their co-twins who are lean, a study setting that distinguishes the acquired features of obesity from potential genetic effects [32]. More functional studies have highlighted lower contents and activities of respiratory complexes and decreased oxygen consumption rates in isolated adipocyte mitochondria [33]. In mice with genetically induced obesity (ob/ob), alteration in mitochondrial mass, morphology and oxidative function of white adipose depots was also observed, which could be ameliorated by rosiglitazone treatment, a strong mitochondrial gene program inducer [34]. Other studies indicated that mice models of obesity display limited mitochondrial oxidative activity, irrespective of the glucose tolerance status [35]. When primary mitochondrial defects are generated in transgenic mice, strong metabolic health responses are observed. In particular, a highly detrimental metabolic phenotype was observed in a condition that mimics obesity-induced ROS elevation, induced by overexpression of mitochondrial ferritin in adipocyte mitochondria [36]. Mitochondria status of adipose progenitors, which are key actors in the control of adipose tissue expansion, also suggests participation in cellular identity and lineage commitment to mature adipocytes, as progenitor mitochondrial dysfunction promotes pro-inflammatory characteristics and inability to get along with differentiation [37]. These and other data reviewed in [38] argue for a strong link between adipose mitochondrial function and obesity-related disorders [39]. Having said that mitochondrial dysfunction is common in obesity-associated diseases, the potential consequence of extracellular mitochondria transfer in metabolic diseases can be considered a friend (with beneficial reinforcement of a functional organelle pool) or a foe (if dysfunctional organelles are being transferred to recipient cells), as discussed in [40].

### 4.2. Adipose Glutamine Metabolism in Obesity

Aberrant metabolism in adipocytes, such as the complex reprogramming of glucose, glutamine and mitochondria metabolism, is considered a hallmark of obesity. Particularly, energy production from substrates like glutamine that can be used exclusively in mitochondria can potentially be affected by changes in organelle density following mitochondria transfer. As the most abundant circulating amino acid, glutamine serves an anaplerotic role by replenishing TCA cycle intermediates for the production of reducing equivalents that drive the mitochondrial respiratory chain and generate lipid. Through additional biochemical pathways, glutamine is involved in the synthesis of glutathione (GSH) and glucosamines [41].

In obese individuals compared to lean subjects, lower glutamine levels were reported in adipose tissue extracts, associated with activated pro-inflammatory pathway [42,43]. In white adipocytes from obese individuals, reduced glutamine levels were found along with increased levels of Uridine Diphosphate N-acetylglucosamine (UDP-GlcNAc) via the upregulation of glycolysis and the hexosamine biosynthetic pathways. In this way, low glutamine promotes nuclear O-GlcNAcylation, a post-translational modification that activates the transcription of pro-inflammatory genes. Conversely, glutamine supplementation of human adipocytes in vitro and in vivo in high-fat diet mice, reversed these effects [43].

Attenuation of adipocyte glutamine levels was linked to reduced glutamine synthetase (*GLUL*) expression in obese adipose tissue in comparison with the non-obese group. Moreover, *GLUL* had a higher subcutaneous adipose expression in non-diabetic normal weight patients than in diabetic normal weight patients [44]. *GLUL* was also closely correlated with weight loss [43]. Given a strong negative correlation between adipose tissue glutamine levels (and *GLUL* expression) and adipocyte volume, it is conceivable that reduced local glutamine levels secondary to adipocyte hypertrophy activate inflammation to promote fat tissue expansion. This response is dynamic; it is reversed once a new state has been achieved and adipose tissue becomes maladaptive in conditions of continuous caloric oversupply.

Low adipose glutamine levels in obesity are particularly compelling, as seminal work by Cheng et al. identified that plasma glutamine and the glutamine-to-glutamate ratio are inversely associated with metabolic risks [45]. In consequence, the potential clinical implication of glutamine supplementation has been studied in a plethora of conditions ranging from critical illnesses [46] to metabolic syndrome [47], type 2 diabetes [48] and COVID-19 [49]. Short-term glutamine supplementation is associated with reductions in body weight and fat mass as well as improved insulin sensitivity and glucose homoeostasis [47]. Moreover, in type 2 diabetic patients, glutamine supplementation results in higher levels of incretins, a group of metabolic hormones that promote insulin secretion [50]. Thus, considering the role of mitochondria in the regulation of adipose tissue remodeling and energy balance [38] and the potential impact of glutamine in regulating mitochondria activity [41], glutamine may constitute an interesting therapeutic compound to reduce obesity-induced adipose tissue dysfunction, a cornerstone in type 2 diabetes pathophysiology.

### 4.3. Adipose Mitochondria at the Center of White/Brown-Like Adipocyte Conversion

Detailed focus on the status of mitochondria in adipose tissue has recently emerged, stimulated by discoveries of phenotypic inter-conversion between so-called “white” lipid-storing adipocytes and its “brown” mitochondria-enriched thermogenic fat cell counterpart. Indeed, brown adipocytes contain a high mitochondria density relative to white adipocytes, which have the unique ability to uncouple oxidative phosphorylation to produce heat, thanks to the specific expression of the mitochondrial Uncoupling Protein UCP1 [51]. UCP1 acts by dissipating the proton gradient through the inner mitochondrial membrane, a process that is otherwise coupled to ATP synthesis. In response to cold exposure or βadrenergic activators, white adipocytes can convert into brown-like fat cells that display a noticeable increase in mitochondria density/activity and thermogenesis [52]. As white adipocytes are just “classical” lipid reservoirs that are filled in proportion to energy excess, thermogenic brown fat cells are capable of heat production, which is an energy-dissipating event. Thus, important physiological consequences are expected for energy homeostasis, depending on the relative proportions of white versus brown-like fat cells in a given subject, likely to influence individual responses to common obesogenic conditions. Therefore, a promising strategy in the fight against obesity is to promote fat cell browning, i.e., the conversion of white into brown-like adipocytes, a process in which mitochondria biogenesis, maintenance and turnover are crucial. In this line, caloric restriction in mice is able to promote brown-like adipocyte recruitment in fat pads, associated with metabolic improvement [53].

The conversion of white fat cells into a brown-like phenotype not only involves induction of UCP1 expression, but also the expansion of mitochondria mass through a specific program governed by key transcription factors. An important one is Peroxisome Proliferator-Activated Receptor gamma 1a (PGC-1a), which is a transcriptional regulator [54] that exerts its actions via interactions with other transcription factors such as Estrogen-Related Receptor alpha (ERRa), Nuclear Factor Erythroid 2-related factor 2 (Nrf-2) and Peroxisome Proliferator-Activated Receptors (PPARs). The identification of PR Domain Containing 16 (PRDM16), another coregulator that binds CAAT/Enhancer Binding Protein beta (C/EBPb) to prevent gene expression associated with either white fat or muscle has also increased understanding of the process of white/brown-like fat conversion [55]. The regulation of mitochondria turnover is also important in the conversion of brown-like to white fat. In particular, the loss of UCP1 expression and whitening of fat upon cessation of thermogenic stimulation is linked to autophagy-mediated mitochondrial clearance that is needed for beige-to-white adipocyte reversal. Indeed, it was demonstrated that inhibition of autophagy maintains functional beige adipocytes even after stimuli withdrawal [56].

From the large range of studies on the process of white to brown-like adipocyte conversion in the fight against metabolic diseases, it remains to be investigated whether mitochondria transfer has some relevance. Do previously white adipocytes acquire extracellular mitochondria to convert into brown-like fat cells? Does the process of fat whitening involve a loss of mitochondria through extracellular extrusion? What is the role of acquisition of mitochondria from non-adipose cell types in the conversion process? These remain unsolved questions that need to be investigated.

## 5. Recent Studies Uncovering the Impact of Mitochondria Transfer from Maladaptive Adipose Tissue

In obesity, overdeveloped adipose tissue becomes maladaptive, with fat cell lipid engorgement, increased fatty acid leak, metabolic inflexibility, endoplasmic reticulum stress and local inflammation leading to insulin resistance [51]. The stress signals initiating such a situation are complex, likely emanating from disrupted metabolism in fat cells as discussed above, but also linked to pro-inflammatory adipose tissue microenvironment and gut microbiota dysbiosis through dietary changes [57]. Mitochondrial dysfunction in obesity-driven adipose tissue pathological remodeling is highly expected to participate in initiation of chronic low-grade inflammation, as well as gut dysbiosis, all of them being considered entry gates of further systemic multi-organ metabolic dysregulation [58]. 

A first clue that mitochondrial extracellular transfer might be a key element in obesity-associated adipose tissue remodeling has come from recognition that adipose tissue is an active site in extracellular vesicle production, which could impact mitochondrial metabolism in adjacent tumors [20]. Indeed, in co-culture systems bringing together a differentiated adipose cell line and melanoma cells, it was observed that adipocyte vesicles could stimulate melanoma fatty acid oxidation by providing both enzymes and substrates. Although the presence of bona fide mitochondria has not been investigated in that study, the link to fatty acid oxidation might suggest a mitochondrial contribution. Direct demonstration of a functional role of mitochondria transfer from adipocytes has now been published [21] and discussed in literature highlights [59]. In this report, an adipocyte-specific mitochondria reporter mouse (MitoFat) was used to establish that local macrophages acquire mitochondria from adipocytes in vivo. Interestingly, this adipocyte-to-macrophage transfer of mitochondria defined a distinct tissue macrophage subpopulation that was found to be reduced in obesity. Further, using a cell-based genome-wide CRISPR-Cas9 screen to search for critical players in macrophage mitochondria uptake, Brestoff and colleagues could identify dependence on heparane sulfate biosynthetic genes (Exostosin, *Ext1* or *Ext2*) which participate in N-Acetyl glucosamine branching of proteins on the cell surface. Genetic deletion of *Ext1* gene in myeloid cells decreased mitochondria uptake by macrophages, increased fat accumulation and reduced glucose tolerance in mice on a high-fat diet. In brief, this demonstrates that adipocyte-to-macrophage mitochondrial transfer has a relevant role in adipose tissue and systemic homeostasis by sustaining M2-like polarization of immune cells, disrupted in diet-induced obesity.

**Figure 1:**
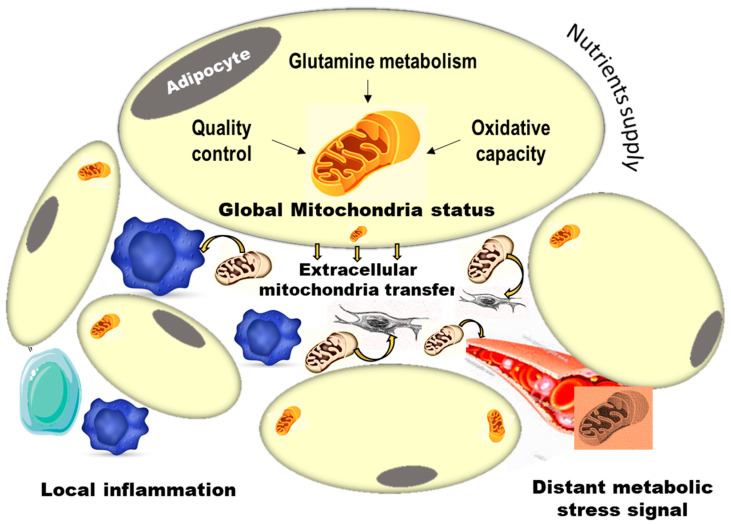
Schematic view of the importance of extracellular mitochondria transfer in adipose tissue metabolic remodeling and systemic metabolic stress signaling. The adipocyte mitochondria global stress status is generated from excessive nutrient supply, defective quality control process and disrupted glutamine metabolism and oxidative capacity of fat cells. This is thought to define the rate of extracellular mitochondria exclusion from fat cells, which is increased in obesity. Subsequent impact of extracellular mitochondria within the adipose tissue micro-environment is expected, by targeting (yellow arrows) immune cells (blue) with consequences on local inflammation, by targeting tissue fibroblasts and progenitors (grey) to orient their fate and differentiation, or by targeting more distant metabolic signaling through the circulation.

More recently, another mouse model using adipocyte-specific overexpression of fluorescent mitochondrial Ferritin confirmed in vivo production of extracellular mitochondria by adipocytes [22]. This study emphasized the importance of mitochondria packaging into extracellular vesicles to convey inter-organ signals. It also demonstrated that adipocyte-derived extracellular vesicles were induced under lipid stress and could protect the heart from ischemia/reperfusion injury. Thus, this study is in line with the idea that the physical transfer of damaged mitochondria is a stress warning pro-oxidant signal relayed from the adipocyte to distant cardiomyocytes. Although more work is needed for a clear picture, an emerging view from these pioneer studies points to an important role for mitochondria exchange in adipose-tissue-driven metabolic homeostasis (Figure 1). 

## 6. Future Directions

The field of extracellular vesicles has contributed evidence of relationships to health and disease conditions, especially in metabolic disorders. Previous studies have revealed changes in circulating extracellular vesicle particle concentrations, in particle density subtypes or in their lipid and protein and miRNA composition when plasma from healthy subjects are compared to patients with metabolic syndrome [60,61], cardiovascular disease [62] or cancer [63]. Extracellular vesicles have been recognized as vectors of biological information by transferring their protein, lipid and nucleic acid content to target cells towards multi-organ responses; however, the idea that they also convey mitochondria, i.e., organelles, is quite new. In agreement, the paper from Crew and colleagues [22] points to high circulating levels of extracellular vesicles containing mitochondrial DNA in unhealthy obese patients compared to healthy obese subjects. In this line, Kayser et al. [64] reported changes in circulating concentrations of phosphatidylglycerols, which are mitochondria-restricted phospholipids, over a wide range of patients from overweight to morbid obesity, with positive association to body mass index. This suggests that circulating mitochondria-containing particles, or even free mitochondria that have been characterized in the bloodstream [65] can be of previously unsuspected importance in the maintenance of metabolic health. Moreover, as mentioned above, it appears so far that a major trigger of mitochondria extrusion from cultured cells relies on the loss of efficiency of cellular quality control systems. In the setting of obesity, autophagy pathways are known to be largely deficient, not only in adipose [66] but also in many other tissues linked to lipid accumulation [67,68,69]. Thus, the obesity context shaped with mitochondrial dysfunction and low autophagy is well matched with a pathologically relevant contribution of extracellular mitochondria transfer that warrants further exploration regarding therapeutic issues.

In the future, it is attractive to imagine that mitochondria could be transplanted in a cell-directed manner as a tool to correct mitochondrial dysfunction in metabolic diseases or other conditions like mutations in mitochondrial DNA. In this regard, although mitochondria can be spontaneously incorporated into cells when added in the culture medium by endocytosis, the transplantation process has low efficiency, and is lacking a cell-specific component in vivo to target incorporation into a particular cell type. Moreover, mitochondria transplantation has to be maintained for a period longer than a few days (which is generally the time frame for evaluation of metabolic changes), particularly when the aim is to correct mutations in mitochondrial DNA. In this regard, sophisticated devices comprising a free mitochondria-containing pipette connected to the cell surface was developed, in which a transient membrane opening was induced by a cavitation bubble formed by a laser pulse generated from an inverted microscope [70]. More recently, a simple-to-use mitochondrial transfer technique called “MitoPunch” was described [71], which consists of a pressure-driven large cargo transfer platform based on cell surgery using a biophotonic laser-assisted photothermal nanoblade. This procedure was shown to be efficient in primary non-immortalized cells in culture to generate a range of defined Mt-DNA/nuclear DNA combinations. These studies provide proof of concept of achievable controlled mitochondria transfer but need further developments towards future in vivo use in therapeutics

## Figures and Tables

**Table 1:** Three different modes of mitochondria transfer have been identified, including direct cell/cell connection by nanotubes, formation of intercellular gap junctions or liberation of extracellular mitochondria-containing vesicles. The table presents a summary of the mode of mitochondria exchange in different cell types from published studies indicated by reference numbers.

Mode of Mitochondria Exchange	Cell Types Involved	Reference Number
Nanotube tunneling	Renal tubular cells	[4]
Intercellular gap junction	Bone marrow stromal cells	[6]
Extracellular vesicles	Mesenchymal stem cells	[7]
Extracellular vesicles	*Caenorhabditis Elegans* neurons	[8]
Extracellular vesicles	Brain (murine, human)	[10]
Not determined	Mesenchymal stem cells	[11]
Nanotube tunneling	Multiple myeloma cells	[12]
Not determined	Osteocytes	[13]
Extracellular vesicles	Autophagy-deficient cell lines	[14]
Extracellular vesicles	Macrophages	[15]
Extracellular vesicles	Mesenchymal stem cells	[16]
Extracellular vesicles	Astrocytes	[17]
Extracellular vesicles	Platelets	[18,19]
Extracellular vesicles	Adipocytes	[20,21,22]
